# Tissue-specific role of dTrmO in threonine decoding during *Drosophila melanogaster* development

**DOI:** 10.1186/s40659-026-00667-0

**Published:** 2026-02-07

**Authors:** Deborah Cuper, Valentina Muñoz-Madrid, Rodolfo Moreno, Francisca Brown, Yueh-Lin Tsai, Álvaro Glavic

**Affiliations:** 1 Departamento de Biología, Centro de Regulación del Genoma, Facultad de Ciencias, Universidad de Chile, Santiago, Chile; 2 New England Biolabs, Inc., Beverly, MA 01915 USA

**Keywords:** tRNA modifications, m^6^t^6^A, TrmO, Translation, Protein homeostasis, Development

## Abstract

**Background:**

Translation is an extremely fine-tuned process. Both speed and fidelity are required to sustain translational demand and protein homeostasis in cells, traits that depend on several elements, including transfer RNA (tRNA) modifications. Additionally, it has been demonstrated that proliferative and differentiated tissues display differential susceptibilities to deficiencies in certain tRNA modifications.

**Methods:**

By using the Gal4/UAS system we manipulated the expression levels of the *Drosophila melanogaster* orthologue of TrmO (dTrmO), a tRNA-methyltransferase that methylates t^6^A in position 37 of tRNAs decoding ACY codons, during development.

**Results:**

Our results show that this protein is necessary for proper translation of ACT codons, and its function is differentially required in proliferative and non-proliferative tissues. Furthermore, we observed that reductions of dTrmO are detrimental for cell growth and proliferation, and muscle function due to impaired translation of Msp300, a nuclear membrane protein specific of muscle fibres. Strikingly, the translation-related phenotypes produced caused by decreased levels of dTrmO in the wing and eye-antenna imaginal disc, fat body, striated muscles, as well as overall organismal growth defects, can be rescued by overexpression of tRNA-Thr-AGT.

**Conclusions:**

Results point to the importance of dTrmO in translation of ACT codons and consequently in cell proliferation and growth of tissues, thus providing further insight about the active role of tRNA modifications in the context of *eumetazoan* development.

**Supplementary Information:**

The online version contains supplementary material available at 10.1186/s40659-026-00667-0.

## Background

Transfer RNAs (tRNAs) were first described as mere adaptors, however during the last 10 to 15 years they have been positioned as key regulators of translation amongst other metabolic processes [1, 2]. Part of their maturation process involves a variety of posttranscriptional chemical modifications [3] in different regions of these molecules, many of them being exclusive to specific isoacceptors. Currently, more than 150 RNA modifications have been discovered, many of them with unknown roles in multicellular organisms [4]. It has been shown the role of tRNA modifications in the anticodon stem loop (ASL) in maintaining accurate translation (fidelity) by stabilizing codon-anticodon interactions, as well as decoding speed, thus allowing correct protein folding and preventing formation of misfolded protein aggregates [5–7]. Loss of some of these modifications lead to a reduced growth phenotype in yeast and other tissue-specific phenotypes in metazoans due to abnormal translation [8–13]. Furthermore, increasing evidence links impaired levels of tRNA modifications to human diseases, from neurological disorders and cognitive disabilities to type II diabetes [14–17], and cancer [18–21].

Previous work in human cell lines described a differential expression of tRNA isoacceptors in proliferative and differentiated tissues related to the use of specific codons (codon bias) in mRNAs expressed and whose functions are associated to proliferation or differentiation of cells [22, 23], while the work of Sagi and colleagues showed variations in the expression levels of a single isodecoder amongst *C. elegans* tissues [24]. Furthermore, tissue specific requirements of tRNA modifications have been observed in flies and mice [25–27]. Changes in tRNA abundance, as well as aminoacylation and modification levels, are a major step in translation regulation under different physiological contexts. For example, mice exposed to hypoxia show tissue specific changes in their tRNA epitranscriptome compared to their normoxic control group [28], whereas the deletion of a tRNA modifying enzyme in *Mycobacterium* is detrimental for its growth in a macrophage infection model [29]. Additionally, budding yeast lacking tRNA 2’O-methyltransferases have an increased sensitivity to oxidative stress [30]. Altogether, these examples support the idea that cells adjust their decoding capacity according to their differentiation state and physiology and highlight the importance of tRNA landscape composition.

First described in *E. coli*, TrmO is a conserved tRNA-methyltransferase that methylates the N6-threonyl-carbamoyl adenosine (t^6^A) moiety present in adenosine 37 in tRNAs that decode ANN codons, resulting in the specific formation of N6-methyl-N6-threonyl-carbamoyl adenosine (m^6^t^6^A) in tRNAs decoding ACY (threonine) codons. An *E. coli* strain deficient in TrmO, thus, with decreased m^6^t^6^A levels, has a reduced capacity to decode ACY codons, as shown by luciferase assays [31]. Importantly, both m^6^t^6^A modification and TrmO are conserved in *eumetazoa* [21, 32].

Studies in *D. melanogaster* have demonstrated that reductions in t^6^A, due to Tcs3 or Tcs5 deficiencies, subunits of the Threonyl-Carbamoyl Transferase Complex (TCTC) which synthesizes t^6^A, in the proliferative cells of the eye primordium impairs its growth, whereas knockdown of these same subunits in postmitotic photoreceptors does not have an effect [33, 34], suggesting a specific translational demand between cells. Since TrmO modifies a subset of threonine decoding tRNAs, and only one of these isoacceptors exists in eukaryotes [35, 36], it was of particular interest to investigate the importance of TrmO in ACT codon translation as a consequence of its methyltransferase activity on tRNA-Thr-AGT, and study how the levels of this enzyme are required for cell proliferation, growth, and development of individuals.

The orthologous gene of TrmO in *Drosophila melanogaster* is registered as *CG12822* (BDGP Rel. 6/dm6) [37]. We evaluated its role in translation of ACT codons and ultimately in development of this organism by manipulating its levels using the Gal4/UAS system [38]. A translational GFP reporter possessing a 5` 4X tandem ACT codons revealed that GFP translation is decreased under loss of dTrmO in wing imaginal discs and the fat body. Strikingly, this reduction of GFP signal can be rescued by tRNA-Thr-AGT overexpression. Further, ACT decoding in an endogenous context, showed that ACC/ACT codon enriched gene *msp300*, necessary for development and functioning of striated muscles [39, 40], was particularly affected by dTrmO knockdown, and this can be also rescued by overexpressing tRNA-Thr-AGT. Finally, dTrmO is necessary for normal organ growth during development, and the decreased growth induced by its deficiency can be reverted by overexpressing tRNA-Thr-AGT as well. Taken together, our results indicate that dTrmO is essential for accurate ACT translation, and this function is likely differentially required for decoding ACT rich genes expressed in specific tissues.

## Materials and methods

### Fly husbandry and stocks

Flies were raised in standard medium (wheat flour 50 g/L, fresh yeast 100 g/L, agar-agar 11 g/L, dextrose monohydrate 80 g/L, propionic acid 6 mL/L and methyl-parabene 1.56 g/L) at 25 °C. Stocks were obtained from the Bloomington *Drosophila* Stock Center and the Vienna *Drosophila* Resource Center. tRNA-Thr-AGT sequence was obtained from the genomic tRNA database (gtRNAdb) [35], synthesized by Genewiz (Genewiz by Azenta Life Sciences, NJ, US) and cloned in the *Drosophila* pHStinger vector along the short RNA promoter u6B sequence. Similarly, 4XACT-GFP was synthesized and cloned by Genewiz in a pUAS-attB *Drosophila* vector. All *Drosophila* transgenic insertions were P-element based done by BestGene (Best Gene Inc., CA, US) except for 4XACT-GFP which were done via ΦC31 integration at attP docking sites in chromosome II (BL8621).

We used the following Gal4 lines in this work: Cg-Gal4 [34], SalP (Sal^EPv^-Gal4) [41], Nub-Gal4 and Ey-Gal4 (gifted by Dr de Celis, Centro de Biología Molecular Severo Ochoa, Madrid, Spain). We also used the following lines from Bloomington Drosophila Stock Center: Tub-Gal4 (BL5138), En-Gal4 (BL30577), Mef2-Gal4 (BL27390), Act5c[FRT.CD2]-Gal4 (for flip-out clones, BL4780) [42], UAS-yellow RNAi (BL64527), UAS-white RNAi (BL33613), UAS-Dcr2 (BL24650), Msp300-GFP (BL59757), Df(2R) BSC265 (BL23164), CG12822[f02022] (BL18503), UAS-mCD8-RFP (BL27391). The UAS-dTrmO RNAi stock (v32029), was obtained from Vienna Drosophila Resource Center [43].

### Larval tissue mounting

Larvae were dissected, fixed, and stained as described by Cruz and colleagues [41]. Tissues were stained with TO-PRO-3 (Invitrogen, 1:200). Additionally, we used anti-phosphorylated histone-3 (1:500, Upstate Biotechnology) and secondary antibody Alexa Fluor anti-rabbit 488 (1:200, Invitrogen). For flip-out clonal analysis, fat body was stained using Rhodamine-phalloidin (1:100, Invitrogen).

For autophagy imaging, fat body was dissected from three hours starved (1% agarose/PBS medium) and normal medium third instar larvae, stained with 50µM Lysotracker DND-99 (Invitrogen) and Hoescht for 30 min, washed with PBS, and mounted in 50% glycerol/PBS.

### Thorax immunofluorescence and mounting

Thorax dissection was performed as described by Weitkunat & Schnorrer [44]. Briefly, samples were fixed in 4% PFA in relaxing solution for 15 min and then washed twice in relaxing solution + 0.3% PBS-Triton X-100, for 5 min each. Thoraces were cut in half using a sharp microtome blade and blocked in BSA 1%/PBS-Triton X-100 0.3% for two hours. Hemi-thoraces were then incubated with anti-green fluorescent protein antibody (Rabbit, Invitrogen #A11122), diluted at 1:2000 in BSA 1%/PBS-Triton X-100 0.3%, at room temperature for one hour, and washed three times with PBS-T 0.3%, for 15 min each. A second blocking step was done afterwards. Samples were incubated with Rhodamine-Phalloidin (1:100 Invitrogen) and secondary antibody (1:200, Alexa Fluor 488 anti-rabbit, Invitrogen) in BSA 1%/PBS-T 0.3% overnight at 4 °C. Nuclei were stained afterwards with TO-PRO-3 (1:200, Invitrogen) for 20 min at RT, and samples were washed three times with PBS-T 0.3% (15 min each wash). Hemi-thoraces were mounted in Vectashield (Vectorlabs), and images were taken using a confocal Zeiss LSM710 Meta microscope.

### Flight assay

Flight assay protocol was made as in [45]. Individual flies were dumped in a 2 L flask. Flies were checked if they were capable of sustaining flight or fell right into the bottom of the flask.

### Flip-out clonal analysis

36 ± 12 h after egg laying individuals were subjected to heat shock (37 °C) for 2 min to induce FLP activity. Third instar larvae with GFP positive cells were treated as described previously.

### Wing and eye morphological analysis

Adult flies fixing was done in ethanol for at least 2 h, and wings were dissected and mounted in a 1:1 lactic acid-ethanol solution. Images were captured using a Nikon SMZ800 stereo zoom microscope, and the Micrometrics SE Premium 4 software. Likewise, for the eye size analysis adults were fixed in ethanol, heads were dissected in cold PBS 1X and then mounted and photographed in methylcellulose. Wings and eyes were measured using ImageJ.

### Image processing and statistical analysis

Image processing was done using ImageJ. Statistical analyses were done using R Studio. All data presented are mean + S.D. and were subjected to t-test or one-way ANOVA, unless stated otherwise in figure captions. p-values lower than 0.05 were considered as significant. Dunn’s multiple comparisons post-hoc tests were used, unless stated otherwise.

### qRT-PCR, four-leaf clover RT-PCR and tRNA-Thr-AGT overexpression

To test RNAi efficiency, total RNA from third instar larvae expressing TrmO RNAi was isolated using TRIzol reagent (Invitrogen), according to the instruction manual, followed by DNase I treatment (New England Biolabs). One microgram of treated RNA was used for cDNA synthesis using Bio-Rad iScript reverse transcriptase mix. qRT-PCR to measure dTrmO expression was performed using primers 5’-GGCCAGCAATAGACTTAGGCGTG-3′ and 5’-CCTCCAGAATGTCCACGATGCAC-3′.

To observe changes in tRNA-Thr-AGT expression among tissues, we used the four-leaf clover qRT-PCR method [46]. Briefly, total RNA samples isolated from thoraces, wing discs and fat body were deacylated, following ethanol precipitation according to [46]. 400 ng of total RNA from each sample were incubated with 80 pmol of the SL-Adapter (5’-Phos TCGTTAGGGTCCGAGGTATTCACGATG – rGrA-3′, Integrated DNA Technologies) in 9 µL for 3 min at 90 °C. 1 µL of 10X Annealing Buffer [46] was added afterwards, followed by incubation at 37 °C for 20 min. 2 µL of 10X Rnl2 enzyme buffer was added, together with 5U (0.5 µL) of Rnl2 enzyme (New England Biolabs), followed by a 2-hour incubation at 37 °C, and a 4 °C incubation overnight. cDNA was synthesized using SuperScript II Reverse Transcriptase (Invitrogen), with RT/R primer 5’-CTAGACAGGCGCTTTAACCAACTAAG-3′ specific for tRNA-Thr-AGT, according to the manufacturer’s protocol. Quantitative Real Time PCR was performed afterwards using RT/R and F primer 5’-GGAGATCGTGAGTTCGAATCTC-3′. 5 S rRNA was used as a normalizer (Fwd: 5′- CCATACCACGCTGAATACATCGG-3′, Rv: 5′-ACGCGGTGTTCCCAAGCG-3′) [47].

For tRNA-Thr-AGT overexpression, a DNA fragment containing the complementary sequence of tRNA-Thr-AGT 1–1 from 3′ to 5’, obtained from the Genomic tRNA Database, flanked by the U6B promoter and terminator (Genewiz by Azenta Life Sciences, NJ, US) was subcloned on the pH-Stinger vector replacing the original Hsp70 promoter and EGFP sequences. We performed the same FL-PCR protocol for measuring tRNA-Thr-AGT levels to confirm the overexpression from larvae RNA samples.

### Tissue-specific codon usage

Transcriptome data for imaginal wing disc, fat body and muscle tissues was retrieved from previous RNA-Seq libraries [48] (See Table S3, Supplementary Data), mRNA reads were aligned using Bowtie2 and were annotated using a GTF file with information from every codon in frame using Rsubread package from R. Data was then normalized and analyzed by DESeq2 [49]. On the other hand, CDS from Fbtr sequences were taken from BDGP6.32.cds.fa (dm6 genome, UCSC repository) and in-house Perl script was set up to reckon ACT/ACC codons and add them up along with the total number of elongator codons for each CDS [50], this program excludes the first codon as the idea is to count only the codons translated by elongating tRNAs and it was used to obtain genome codon frequency and codon usage. Using a list of differentially expressed genes we obtained the corresponding sequences for each tissue and filtered repeated transcripts, selecting the longest one for analysis. Therefore, by multiplying normalized transcript count obtained by DESeq2 and CDS codon frequency, we obtained the codon usage for each CDS. Normalization was performed by dividing the summed usage of each codon by the maximum sum across all 64 codons, scaling the values between 0 and 1. This approach facilitated direct comparisons across datasets. Codon usage bias was calculated comparing codon usage for each tissue regarding its usage in the genome; to estimate differential codon usage we used a ratio for reference. Data analysis was conducted using R software environment (4.4.1 version).

### LC-MS/MS

1 µg of total RNA extracted from *Drosophila* treated with control and TrmO RNAi were digested to nucleosides at 37$\:^\circ\:$C overnight using a Nucleoside Digestion Mix (NEB, Cat #M0649S). The digested RNAs were subsequently injected without prior purification on an Agilent 1290 Infinity II UHPLC equipped with a G7117 diode array detector and an Agilent 6495 C Triple-Quadrupole Mass Spectrometer operating in positive electrospray ionization (+ ESI) mode. UHPLC was conducted on a Waters XSelect HSS T3 XP column (2.1 × 100 mm, 2.5 μm) containing methanol and 10 mM ammonium acetate (pH 4.5) gradient mobile phase. Mass spectrometric data were acquired using dynamic multiple reaction monitoring (DMRM) mode. Each nucleoside species was identified based on the associated retention time and mass transition in the extracted chromatogram. Mass transition monitored: m^6^t^6^A (427.1 -> 295.2); Ψ (245.1 -> 209.1). m^6^t^6^A level was estimated by normalizing its mass response to that of Ψ.

### Western blot experiments

Larvae were homogenized mechanically in lysis buffer for protein extraction (PMSF 0.1 mM, HEPES 40 mM pH 7.5, NaCl 100 mM, Sodium Pyrophosphate 10 mM, Beta-glycerolphosphate 10 mM, Sodium Fluoride 50 mM, CHAPS 0.3%, Halt Protease Inhibitor Cocktail 1X by Thermo Scientific, and EDTA 0.1 mM). Samples were separated using a 12% polyacrylamide gel by electrophoresis. Trichloroethanol (Sigma Aldrich by Merck) staining was performed using a UV transilluminator. Proteins were transferred to a nitrocellulose membrane (Amersham protran 0.2 μm nitrocellulose membrane, Merck). Membranes were blocked using 5% BSA/TBS-Tween 20 0.1% overnight. To detect *Drosophila* dTrmO, membranes were incubated with a rabbit polyclonal antibody targeting human TRMO (1:250, 1% BSA/TBS-T 0.1%, Atlas antibodies HPA021281), overnight at 4 °C. Membrane was washed three times with TBS-T 0.1% and incubated with goat anti-rabbit HRP conjugated secondary antibody was used (1% BSA/TBS-T 0.1%, 1:2500, Merck 12–348, Germany) for two hours at room temperature, followed by three washes with TBS-T 0.1%. Chemiluminiscencent signal was revealed using SuperSignal West Femto (Thermo Scientific).

## Results

We identified the ortholog gene of *E. coli* TrmO in *D. melanogaster* registered as *CG12822* by using NCBI BLAST tool [51]. Like *E. coli* TrmO, CG12822-PA predicted protein structure has a TsaA-like domain, which belongs to Yae-B superfamily, and a central S-adenosyl-methionine binding site (InterPro) [52, 53] (Fig. S1). Moreover, protein sequence alignment between *E. coli* and *D. melanogaster* orthologues shows conservation of amino acids of the active site as reported by Kimura and colleagues [31], together with an extra domain of approximately 100 amino acids at the N-terminus, which is also absent in human ortholog TRMO [54] (Fig. S1A, green boxes). *CG12822* has two annotated transcripts, and two protein isoforms (Fig. S1B), and is predicted to have tRNA-methyltransferase activity (Fig. S1C). Moreover, western blot experiments using antibody for human TRMO targets an overexpression construct of CG12822 isoform A tagged to 3xFLAG epitope (Fig. S1E). Thus, due to its structural and likely functional conservation we will refer to this gene as *dTrmO*.

dTrmO is required for efficient translation of ACT codon translation in wing disc and fat body cells.

**Fig. 1:**
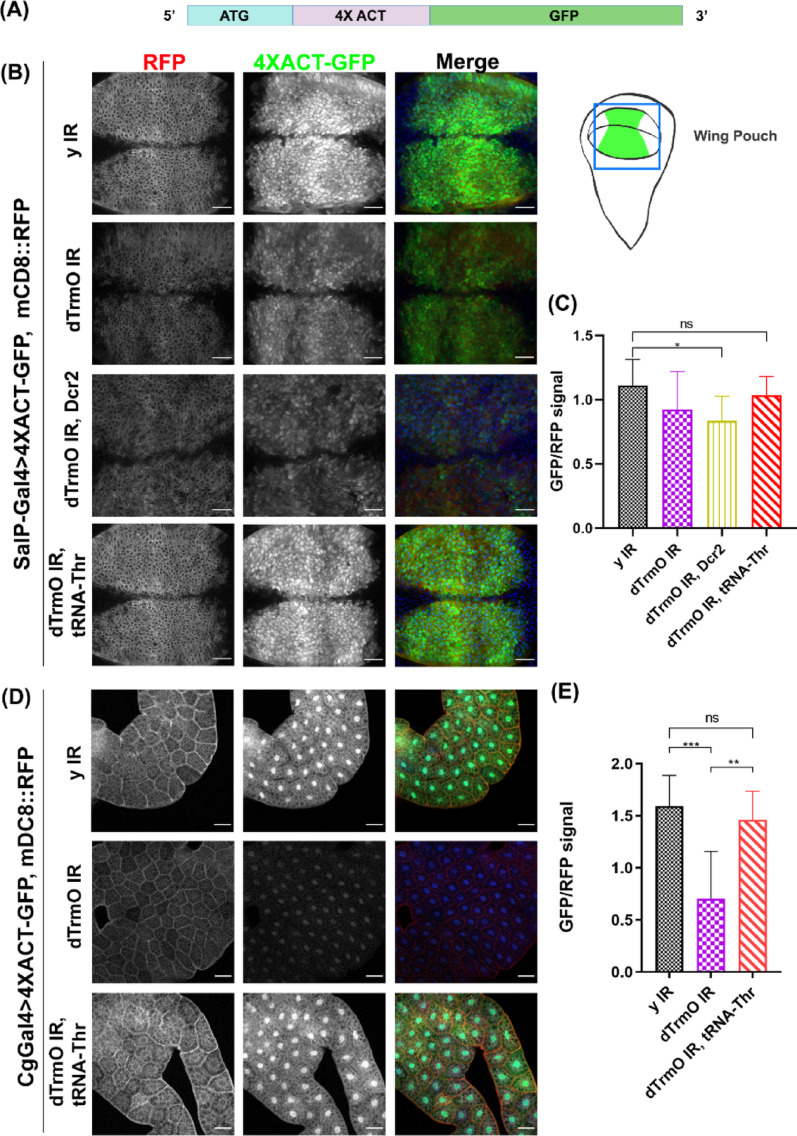
ACT enriched translational GFP reporter levels in the wing imaginal disc. **A** Schematic of the GFP reporter construct. Four ACT codons are located downstream of the translation initiation site and upstream of GFP coding sequence. **B** ACT codon enriched GFP together with mCD8::RFP overexpression in wing discs using the Gal4 driver SalPe [41], genotypes indicated for each condition. RNAi targeting y (y IR) is used as a control. Scale bar = 20 μm. (Right) Schematic of a wing imaginal disc. Square shows the wing pouch compartment, which forms the wing blade, with the SalP expressing territory in green. **C** One-way ANOVA of GFP/RFP ratio. Dunn’s multiple comparisons test shows a significant difference of GFP signal under dTrmO knockdown along with Dcr2 overexpression. RNAi overexpression against dTrmO together with tRNA-Thr-AGT overexpression shows a non-significant change in GFP/RFP levels. p-value < 0.05. *n* = 12, 16, 11 and 11 discs for each condition, respectively, from three independent crosses. **D** Larval fat body mounts of animals expressing the translational GFP reporter combined with mCD8::RFP as a control. Genotypes are shown in each row. Nuclei stained with TO-PRO (blue). *n* = 11, 10 and 13 ROI of different fat body images for each condition, respectively, from two independent crosses. Scale bar = 50 μm **E** One-way ANOVA of a ROI GFP signal normalized by its RFP signal for each condition, p-value < 0.05. Dunn’s multiple comparisons test shows significant differences between controls (y IR) and dTrmO knockdown (p-values < 0.05)

Considering the conservation of this enzyme across evolution, we assessed if dTrmO levels influence proper ACT translation in *D. melanogaster*. Since no canonical ACC decoding tRNA (tRNA^Thr^_GGT_) exists in this species [35, 36], we designed an ACT codon enriched GFP translational reporter (Fig. 1A) and induced its expression using the Gal4/UAS system in proliferative cells, such as the wing imaginal disc or in non-proliferative cells, e.g. the fat body, to address the impact of the TrmO-dependent modification in tissue-specific translation. Inducible expression of a dsRNA targeting both dTrmO isoforms (Fig. S1B, RNAi efficiency in Fig. S1D, also shown in western blot to measure dTrmO levels, S1E) in wing discs using the SalP-Gal4 driver decreases GFP signal in this proliferative tissue, effect that is further enhanced by its overexpression with Dicer-2 (Dcr2) protein, which is part of the RNAi processing pathway [43] (Figs. 1B and C, S2). Additionally, this reporter expressed in the fat body using Collagen IV (Cg) Gal4 driver shows decreased levels of GFP under dTrmO knockdown condition (Fig. 1D and E). To establish if impaired ACT decoding is due to deficient decoding capability of tRNA-Thr-AGT [55, 56], we combined dTrmO knockdown with the overexpression of tRNA-Thr-AGT using the RNA Polymerase III dependent U6B promoter (see materials and methods, Fig. 1B and E, S3). This recovers GFP signal under reduced dTrmO activity in both tissues, suggesting that impaired GFP translation is likely due to inefficient decoding of ACT codons. Since dTrmO mRNA is produced by splicing of Atg10 primary transcript, it is worth highlighting that we ruled out possible effects on autophagy and Atg10 activity (Fig. S4) as a confounding indirect effect.

### dTrmO is required for normal muscle development due to its role in ACT/ACC codon decoding in *D. melanogaster*

**Fig. 2:**
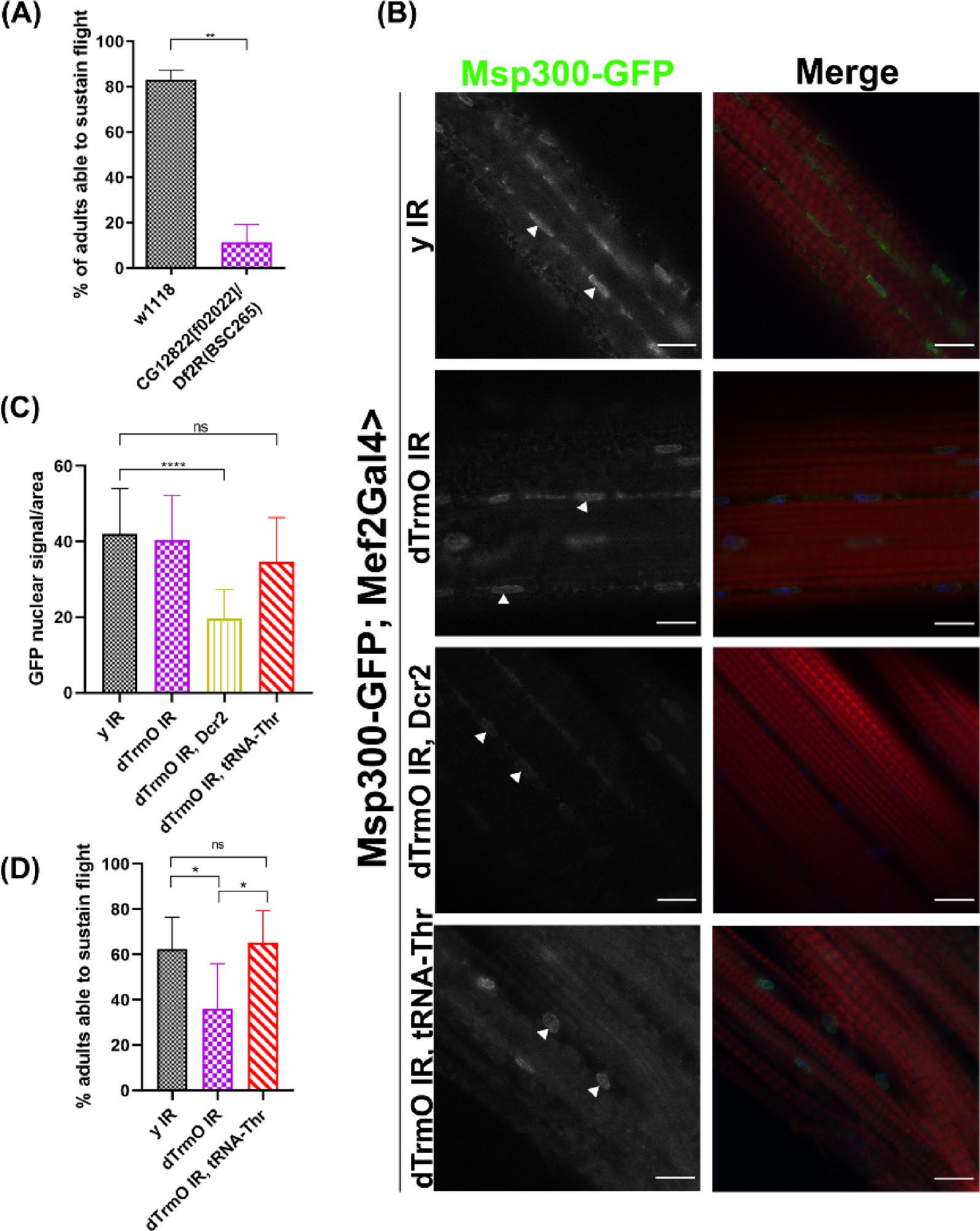
ACT decoding of the ACT/ACC codon enriched gene *msp300*. **A** One-way ANOVA of flight capacity of control (w^1118^) and dTrmO mutant animals (CG12822[f02022]) crossed to a chromosomal deficiency spanning *dtrmO* locus (Df(2R)BSC265). Flies were individually tested, and each condition was measured in groups of 20–40 animals. p-value < 0.05. **B** Thorax immunostaining against GFP shows a decrease of Msp300-GFP protein signal in muscle nuclei (arrowheads) under dTrmO knockdown and overexpression of Dcr2 in these cells. Scale bar = 10 μm. Stained with TO-PRO-3 (blue) and phalloidin (red). (**C**One-way ANOVA of GFP fluorescence intensity of muscle fibre nuclei normalized by area, *n* = 25, 29, 30, and 32 nuclei for each condition respectively, p-values <  0.05. **D** Prop-test of flight capacity under different Mef2-Gal4 induced knockdown, using RNAi against yellow (y) as a control, shows that tRNA-Thr-AGT overexpression rescues dTrmO knockdown phenotype. p-values obtained by Bonferroni’s adjustment method, p-values <  0.01

Considering our previous results, using a bioinformatic tool that counts codons in all *D. melanogaster* CDS and normalizes this by the total number of codons within each sequence, estimating the frequency for each CDS, we sought for ACT codon enriched genes that could be affected due dTrmO deficiency and therefore impaired in translation (Table S1). One possible candidate was the muscle specific protein Msp300, whose function is related to proper muscle nuclei positioning and establishment of the neuromuscular junction in striated muscles [39, 40, 57]. Interestingly, in a previous work [58], aimed to identify genes required for muscle function in *D. melanogaster*, it was reported that knockdown of dTrmO using the muscle Gal4 driver, Mef2, generates a “flightless” phenotype, suggesting an impaired function of striated flight muscles under dTrmO deficiency. This phenotype was confirmed in dTrmO hemizygous mutants as shown in Fig. 2A. In accordance, by using a fly strain expressing Msp300 fused to GFP [59, 60], we observed that Msp300-GFP nuclear signal in adult thoraces was diminished under dTrmO muscle knockdown condition and importantly, this reduction was also rescued by tRNA-Thr-AGT overexpression (Fig. 2B, arrowheads, and 2C).

To test if impaired flight muscle function produced by reduced dTrmO was due to inefficient ACT/ACC decoding and thus *msp300* translation, we measured flight capacity in animals expressing the dTrmO RNA interference construct in flight muscles and in animals additionally overexpressing the tRNA-Thr-AGT. Figure 2D shows a decreased flight capacity of dTrmO knockdown animals, and this trait is effectively restored upon overexpression of tRNA-Thr-AGT.

### dTrmO is essential for cell proliferation during development

**Fig. 3:**
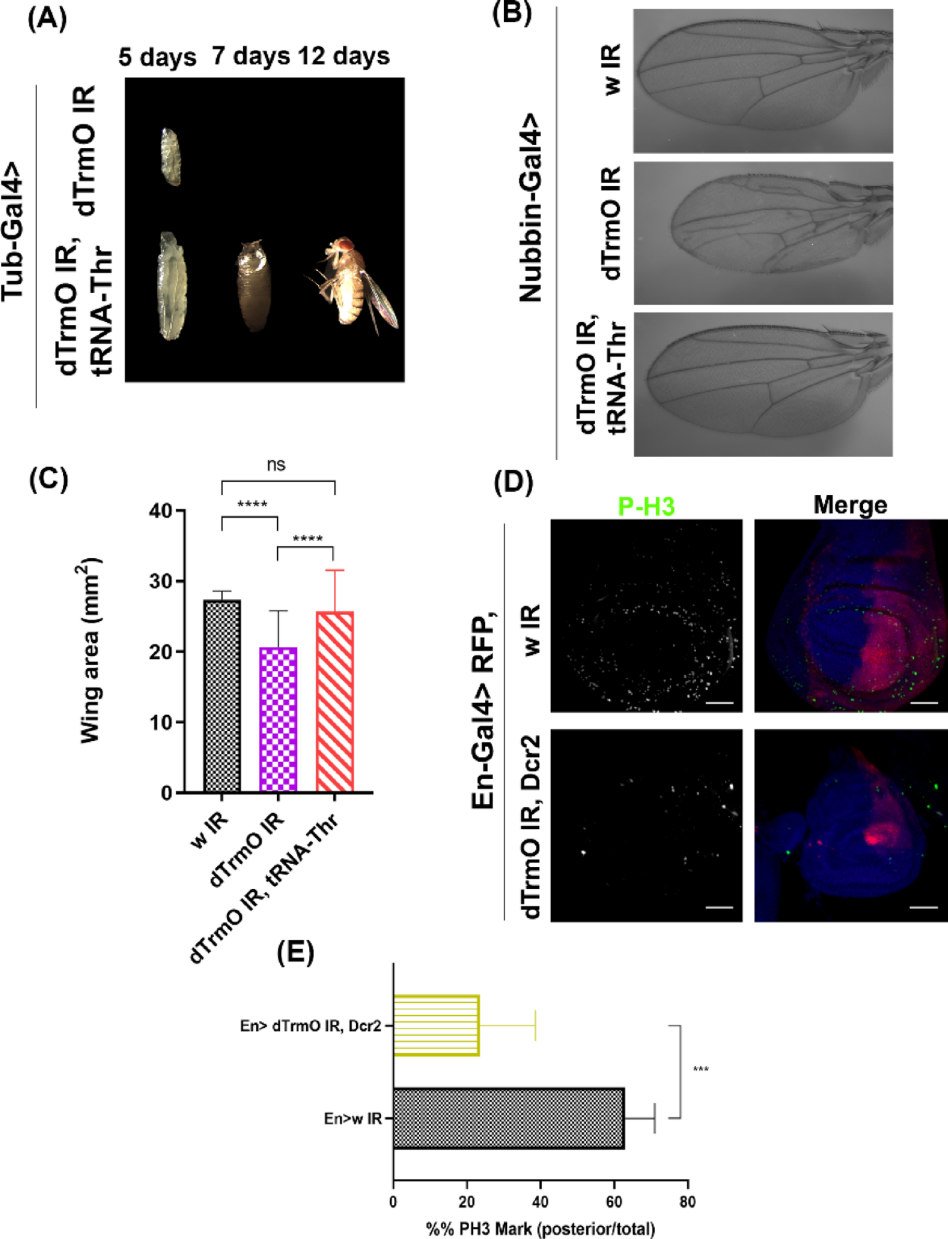
dTrmO knockdown is detrimental for individual growth. **A** 5-, 7- and 12-days old animals of dTrmO knockdown in the whole animal. Bottom condition shows rescue of growth upon overexpression of tRNA-Thr-AGT. **B** Wing mounts of females expressing Nubbin-Gal4 driver (wing blade), with RNAi against *white (w)* as controls. **C** One-way ANOVA of wing blade area. Tukey’s multiple comparison test shows a significant decrease of wing area upon dTrmO knockdown (p-value < 0 .05), which is recovered in dTrmO IR, tRNA-Thr-AGT conditions (p-value >  0.05, *n* = 103, 181 and 155 wings for each condition, respectively, from three independent crosses). **D** Immunofluorescence against phosphorylated histone 3 under control (white IR) and dTrmO knockdown conditions in the posterior compartment of wing discs using En-Gal4 driver. Scale bar = 50 μm. **E** Unpaired t-test shows a significant decrease of phosphorylated histone 3 mark in the posterior compartment under dTrmO knockdown conditions. p-value <  0.05, *n* = 4 and 7 discs for each condition, respectively

To characterize other phenotypes originated by reductions in dTrmO function during development, we first knocked down this gene in the whole animal. Figure 3A shows that dTrmO knockdown larvae have an extremely reduced size and die before pupariating (5 days old larvae). Strikingly, developmental progression can be rescued by overexpressing tRNA-Thr-AGT (Fig. 3A), suggesting that dTrmO knockdown lethality is likely due to impaired ACT decoding.

According to Gingold and colleagues [22], a differential codon usage exists between proliferative and differentiated cells in human cell lines, which is related also to a differential expression between isoacceptors amongst these cells. Codon usage bias has also been observed in other biological systems, including different prokaryotes [61, 62], fission yeast, *C. elegans*,* X. laevis*, and *Drosophila* [63]. Four-leaf clover qRT-PCR experiments show that there is indeed a differential expression of tRNA-Thr-AGT between wing discs, thoraces, and fat body, with a higher relative expression in the latter (Fig. S5), while tissue specific qRT-PCR assays suggest that the adult thorax has the highest expression of dTrmO, while the fat body has the lowest (Figs. S6 and S7). Additionally, we observed a bias in ACT and ACC codon usage in genes differentially expressed in these tissues (Table S2). Our analysis shows an increased frequency of these codons in larval muscles, fat body, and wing disc transcriptomes compared to the *D. melanogaster* genome, suggesting an enrichment for these codons during translation (Table S2). Considering this, we assayed tissue-specific dTrmO knockdown outcomes in *D. melanogaster* larvae. Figure 3B and C highlight a significant decrease in wing size and mild wing pattern abnormalities under dTrmO knockdown conditions. Furthermore, immunofluorescence against phosphorylated histone 3 (Fig. 3D) shows a diminished cell proliferation under dTrmO reductions (Fig. 3E), suggesting that this enzyme is necessary for cell proliferation in the wing primordia and therefore required to sustain normal development of this tissue. As in our previous results, the detrimental effect on growth in this highly proliferative tissue was also suppressed by overexpressing tRNA-Thr-AGT.

### The function of dTrmO in the fat body allows normal animal growth

**Fig. 4:**
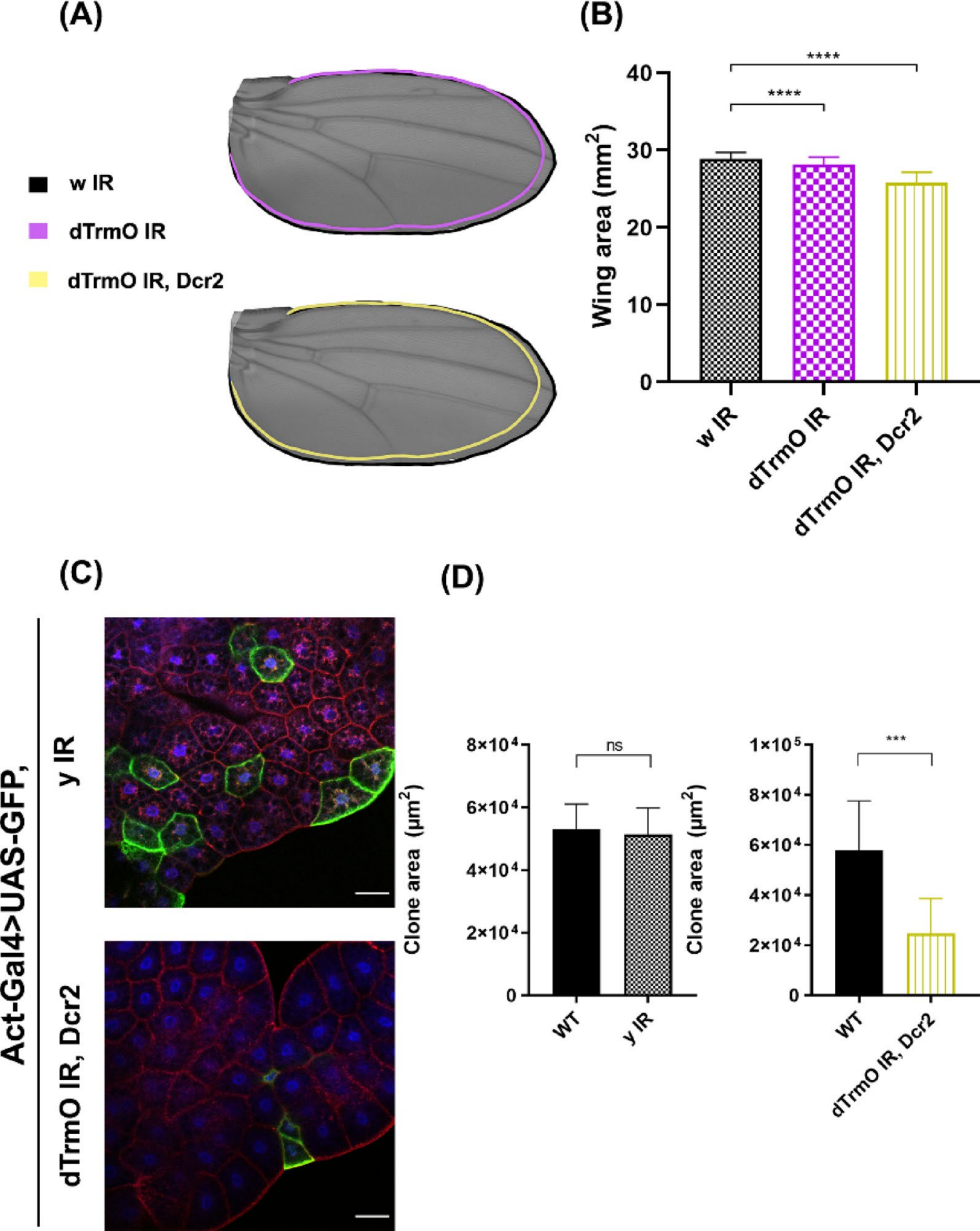
Tissue specific dTrmO knockdown in the fat body. **A** Comparison of wing size among dTrmO loss and gain of function to a negative control. Each condition is highlighted in its respective colour. **B** One-way ANOVA shows significative difference among groups, and Dunnett’s multiple comparison test establishes differences between dTrmO RNAi and dTrmO RNAi combined with Dcr2 overexpression (p-value <  0.05), *n* = 108, 107, and 140 wings, from three independent crosses. **C** Fat body mounts of yellow (y) IR and dTrmO IR, Dcr2 cells (both GFP positive), stained with Phalloidin (red) and TO-PRO-3 (blue). Scale bar = 50 μm. **D** Cell size unpaired t-test shows no significant difference between y IR cells (6 cells) and normal neighbouring WT cells (28 cells) (top), and a significant difference between neighbouring WT (32 cells) and dTrmO IR, Dcr2 positive cells (7 cells) (bottom), p-value <  0.05

The fat body is an important organ that regulates organismal growth during *D. melanogaster* development. It is a secretory tissue that produces several hormones and structural proteins like collagen among others, therefore, its role as a growth regulator strongly depends on accurate translation and protein homeostasis [64]. Cells in the fat body do not proliferate and their growth depends on endoreplication, thus is a model of non-proliferative tissue. Loss of dTrmO function in this tissue decreases adult wing size (Fig. 4A). This effect is further enhanced by the overexpression of Dcr2. Additionally, to explore cell-autonomous effects of dTrmO deficiencies in fat body cells, we randomly induced yellow (control) or dTrmO knockdown cells by using FLP-FRT mediated flip-out clones in the developing fat body (shown as GFP positive cells) [42]. Figure 4C and D show a decreased cell size of dTrmO RNAi expressing cells compared to their WT neighbouring cells, therefore demonstrating the importance of this enzyme in the fat body to support cell growth autonomously.

### dTrmO is required to sustain proliferation in the eye-antenna imaginal disc, but not photoreceptor differentiation

**Fig. 5:**
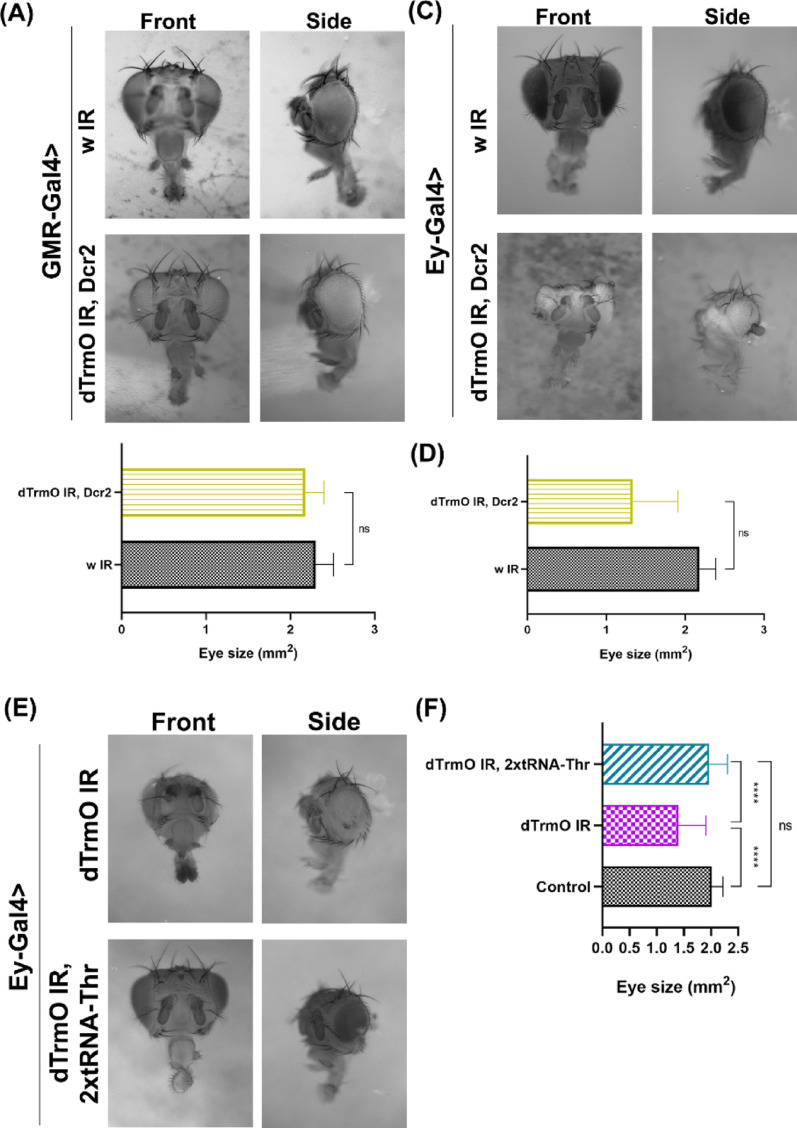
Loss of function of dTrmO in the proliferative and differentiated compartment of the eye-antenna imaginal disc. **A** Front and side view of adult heads expressing dTrmO RNAi in the differentiated cells of the eye disc (GMR-Gal4). Side view: Anterior (left), posterior (right), dorsal (top), ventral (bottom). **B** Unpaired t-test shows no statistically significant differences between tested groups (p-value >  0.05, *n* = 24 and 28 eyes for each group respectively). **C** Front and side view of adult heads of dTrmO knockdown in the proliferative territory of the eye disc (Ey-Gal4). Arrowheads highlight ectopic structures appearing in the eye. Side view: Anterior (left), posterior (right), dorsal (top), ventral (bottom). **D** t-test shows difference (p-value <  0.05, *n* = 45 and 38 eyes) among both groups. **E** dTrmO RNAi and dTrmO RNAi with two extra copies of tRNA-Thr-AGT adult heads. **F** One-way ANOVA shows a significant increase of eye size under dTrmO knockdown conditions together with two copies of tRNA-Thr-AGT, (p-values <  0.05, *n* = 21, 29 and 33 eyes for each condition, respectively)

To extend our observations to other proliferative tissues in the larvae we further studied the loss of dTrmO in the eye-antenna imaginal disc. During the third instar larval stage, this tissue has both, a proliferative and a postmitotic cell population, thus serving as a good model to study the differential requirement of this enzyme in proliferative and non-proliferative cells in the same organ [65]. Considering this, we reduced dTrmO levels in each territory independently (Fig. 5). Knocking down dTrmO in the differentiating photoreceptors (GMR-Gal4) did not generate changes in eye size or any other evident external alterations (Fig. 5A and B). However, when dTrmO was knocked down in the proliferative region of the eye disc (Ey-Gal4), adults developed smaller eyes with abnormal ommatidia patterns, as shown in Fig. 5C and D. Likewise previous phenotypes described, this phenotype is rescued also by tRNA-Thr-AGT overexpression (Fig. 5E and F), therefore suggesting that like previous results, dTrmO is necessary in proliferating cells to sustain the proper ACT codon decoding requirements that support eye development.

## Discussion

Transfer RNAs are non-coding RNAs first described in 1958 [66] as key players of translation in all living beings due to their role as adaptors. During translation, tRNAs deliver the matching amino acids to the nascent polypeptide chain by pairing with codons read by the ribosome according to the genetic code [67]. However, by changing their physical-chemical properties, tRNA modifications have been shown to fine-tune translation of cells modifying translational accuracy and speed [5, 6, 68], thus representing a different level of regulation in the translation process. Moreover, loss of these modifications have been implied in altered protein homeostasis of cells [8], reduced cell growth capacity [69], increased sensitivity to stress [55, 70, 71], and diseases [15, 20, 72].

Animal development is an extremely regulated and dynamic process. During this process, intracellular and extracellular signals induce changes in protein synthesis according to cell fates. Thus, tRNA modifications represent a subtle mechanism that permits cells to optimize protein synthesis according to their translational demand, a characteristic of their identity and function. Interestingly, it was demonstrated in human cell lines that proliferative and differentiated cells have a distinct codon usage, which correlates to tRNA availability, thus helping cells “optimize” translation [22], a correlation that has also been observed throughout development in mice [26]. An example of a differentially required tRNA modification during development is t^6^A [33, 34], which is necessary for development of proliferative cells of the eye imaginal disc, but not in the differentiating photoreceptors. Furthermore, translational defects specific to β-pancreatic cells in mice due to loss of the tRNA modifying enzyme CDKAL1 has been reported [12]. In addition, tRNA modification Q (queuosine) levels in *Drosophila* were demonstrated to change during development [73], therefore not only suggesting the importance of both, a specific demand for isoacceptors among tissues and the existence of a modified tRNA pool that allows optimization of translation according to cell identity.

This work focuses on the *D.melanogaster* TrmO ortholog, dTrmO, and its role in translation and tissue development. Initially, we characterized the function of dTrmO in ACT decoding and found that loss of this enzyme impairs translation of an ACT-enriched reporter in wing imaginal discs and fat body cells, and importantly, the translation of the ACT-enriched muscle-expressed gene *msp300*. Strikingly, GFP signal and flight phenotype can be restored upon overexpression of tRNA-Thr-AGT, suggesting that impaired translation is due to inefficient ACT decoding. Additionally, knocking down dTrmO in the wing disc reduces wing size due to abnormal cell proliferation, possibly related to dTrmO function in ACT translation optimization, mechanism that appears to be necessary to sustain the cell cycle. In accordance with the general impairment of ACT codon decoding produced by dTrmO deficiency, all phenotypes analysed were reverted by overexpressing tRNA-Thr-AGT, indicating the selective demands of this decoding process in specific genes and cell processes, as well as the function of this protein in the modification of the ACT decoding tRNA.

Additionally, tissue-specific RNA-Seq data [48] (Fig. S6) and qRT-PCR analysis (Fig. S7) suggest that the fat body has lower dTrmO expression in comparison to wing discs and thorax samples, whereas FL-PCR experiments of tRNA-Thr-AGT show an increase by several times of this tRNA in the fat body samples, that could possibly compensate the high translational demand of ACT/ACC codons in this tissue (Fig. S5). Furthermore, it is necessary to remark that human TRMO was described to modify tRNA^Ser^_GCT_. Considering this, we cannot rule out the possibility of the hypomodified tRNA^Ser^_GCT_ having an effect in our results, however the significant reversal of the phenotypes produced in dTrmO knockdown conditions by tRNA-Thr-AGT overexpression strongly support our interpretations.

Given that the fat body is a non-proliferative organ with a high translational demand, it was our interest to test the role of dTrmO in function of this tissue. Flip-out clonal experiments show that dTrmO is required for cell autonomous growth, and appropriate function of this tissue, since knockdown of this gene decreases adult size. An interesting point to address in the future to explain this systemic effect would be the effect on the secretion of insulin-like peptides (dILPs) by fat body cells or by the insulin producing neurons, cells that integrate the nutrient status with organismal growth. These hormones regulate several aspects of *Drosophila* physiology, including growth [74, 75].

The eye imaginal disc has both proliferative cells and post mitotic photoreceptors, thus being an appealing model to study loss of dTrmO in both cell types in the same organ. Considering our results in wing imaginal discs it was anticipated that reductions of dTrmO in proliferative cells of the developing eye leads to a diminished size of this organ in adults. This is rescued also by overexpression of tRNA-Thr-AGT. However, this does not apply when knocking down dTrmO in the differentiating portion of the eye, further indicating that cells have a differential translational requirement depending on their identities. We propose that proliferating cells, due to their required *de novo* tRNA synthesis, are more sensitive to dTrmO deficiencies, as any residual dTrmO expression and modified tRNAs prior to tissue specific knockdown will likely get diluted.

Further questions arise from our results. Although dTrmO was described as an enzyme that modifies both ACC and ACT decoding tRNAs in *E. coli* [31], interestingly, no tRNA^Thr^_GGT_ is present in *D. melanogaster*, nor in vertebrates [35, 36], however it has been described that tRNA-Thr-AGT can be subject of adenosine to inosine editing at position 34, by ADAT2/3 in eukaryotes, expanding the decoding capacity of this tRNA [76, 77]. Moreover, the screening performed by Schnorrer and colleagues [58] shows that knockdown of dADAT2 in muscles gives rise also to a “weak flier” phenotype. Since *msp300* is enriched in both ACC and ACT codons, this gene is also a good candidate to use as a proxy to assess ACC/ACT dual decoding capacity of tRNA-Thr-AGT and its relation with the different levels of dTrmO and dADAT2/3 [78]. Furthermore, we ruled out the possibility of changes in stability of the tRNA, since FL-PCR of dTrmO knockdown animals do not show major changes in steady-state of this tRNA (Fig. S8).

Since changes in tRNA modification profiles help maintaining protein homeostasis through optimization of translation under stressful conditions [70, 81–83], another interesting aspect to consider for future investigations is analyse the role of dTrmO in stress tolerance and response to environmental cues of animals. Furthermore, the formation of tRNA-derived fragments (tRFs) can be another interesting phenomenon to consider, due to tRNA modifications serving as site recognition sites for cleavage, and the role of tRFs in modulation of translation as part of a stress response [84, 85].

Finally, our work suggests that cell type specific expression levels of tRNAs the enzymes responsible of their modifications could provide a platform to support the changes in protein synthesis that sustain and give robustness to the developmental process during the acquisition of specific cell fates.

## Supplementary Information

Below is the link to the electronic supplementary material.

Supplementary Material 1

Supplementary Material 2

Supplementary Material 3

Supplementary Material 4

## Data Availability

Not applicable.
