# Supplementary material for: Tissue-specific role of dTrmO in threonine decoding during Drosophila melanogaster development
Source: Biol Res. 2026 Feb 7;59:16. doi: 10.1186/s40659-026-00667-0 (PMC12977680; doi:10.1186/s40659-026-00667-0)
Supplement: Supplementary file 4 — Supplementary Material 4 [file 40659_2026_667_MOESM4_ESM.docx]

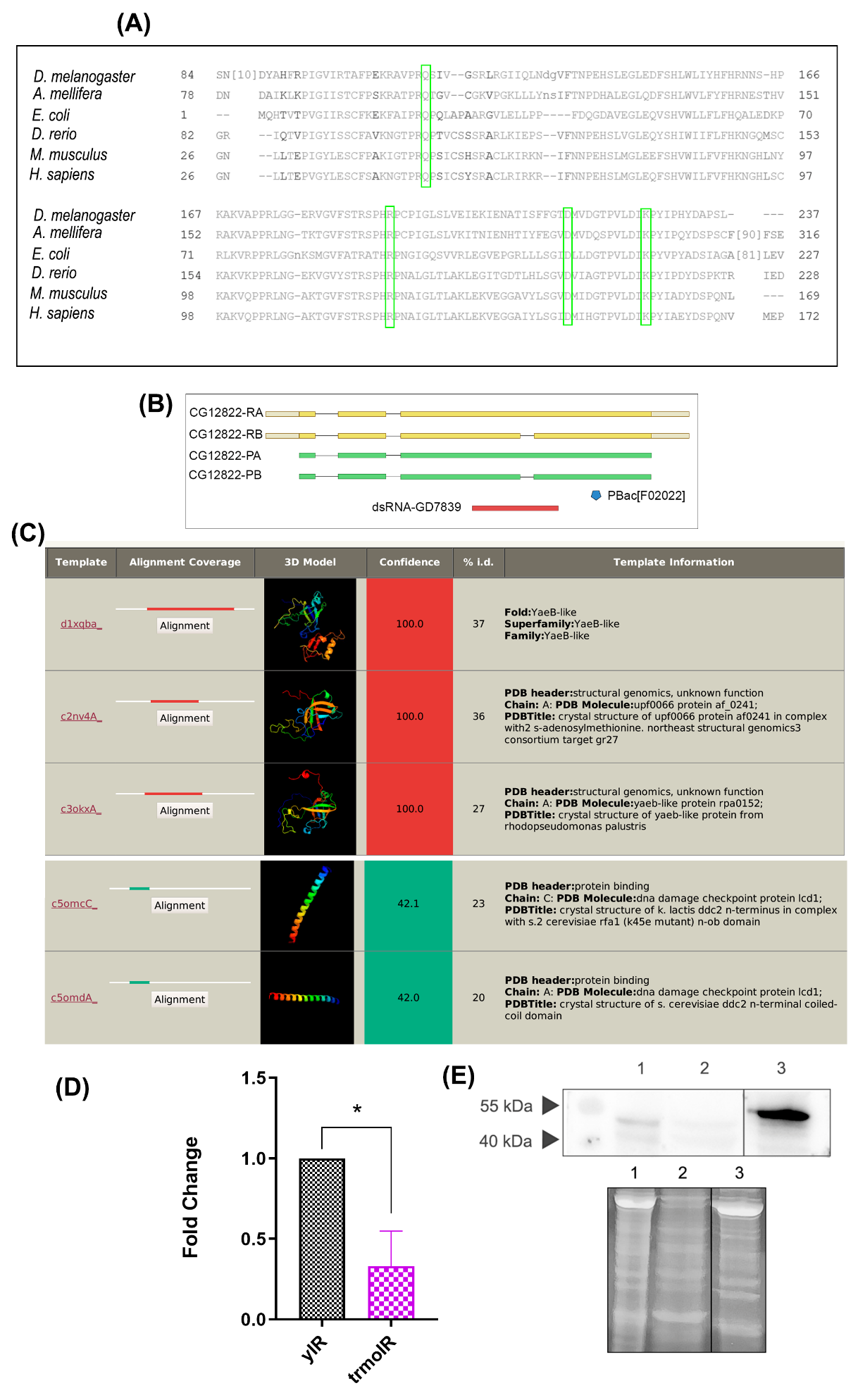


Figure S1: *Drosophila melanogaster* CG12822/dTrmO is a conserved enzyme. (A) COBALT Protein sequence alignment of *dTrmO,* with its orthologs in *A. mellifera*, *E. coli*, *D. rerio*, *M. musculus* and *H. sapiens*. Green rectangles show conservation of active site residues described for *E. coli* TrmO. (B) *CG12822* has two isoforms in *D. melanogaster*. dsRNA used against *CG12822* in this work targets both isoforms, as shown by the red rectangle. Blue triangle highlights the position of the *CG12822/dTrmO* mutant strain used. (C) *Drosophila melanogaster* dTrmO tertiary structure predicted model using Phyre2 (Kelley et al., 2015) shows a 37% identity match with the YaeB/TrmO-like protein family, and a 36% and 27% identity match with the crystal structures of *E. coli* TrmO homologues from *Archaeoglobus fulgidus* and *Rhodopseudomonas palustris*, respectively. *Drosophila melanogaster* dTrmO tertiary structure predicted model using Phyre2 (Kelley et al., 2015) shows no homology with previously characterized crystal structures for the N-terminal of dTrmO. (D) qRT-PCR experiments show a decrease in dTrmO expression using RNAi v32029. (E) Western blot experiments using an antibody targeting human TRMO. Lane 1 is y IR (controls), lane 2 is dTrmO IR, and lane 3 is UAS-dTrmO-FLAG. Two isoforms matching the molecular weight of CG12822 appear, while lane 3 has a strong band in a higher weight, highlighting the overexpression of dTrmO tagged to a 3xFLAG epitope (~57 kDa). Bottom image is trichloroethanol staining, used for loading control.


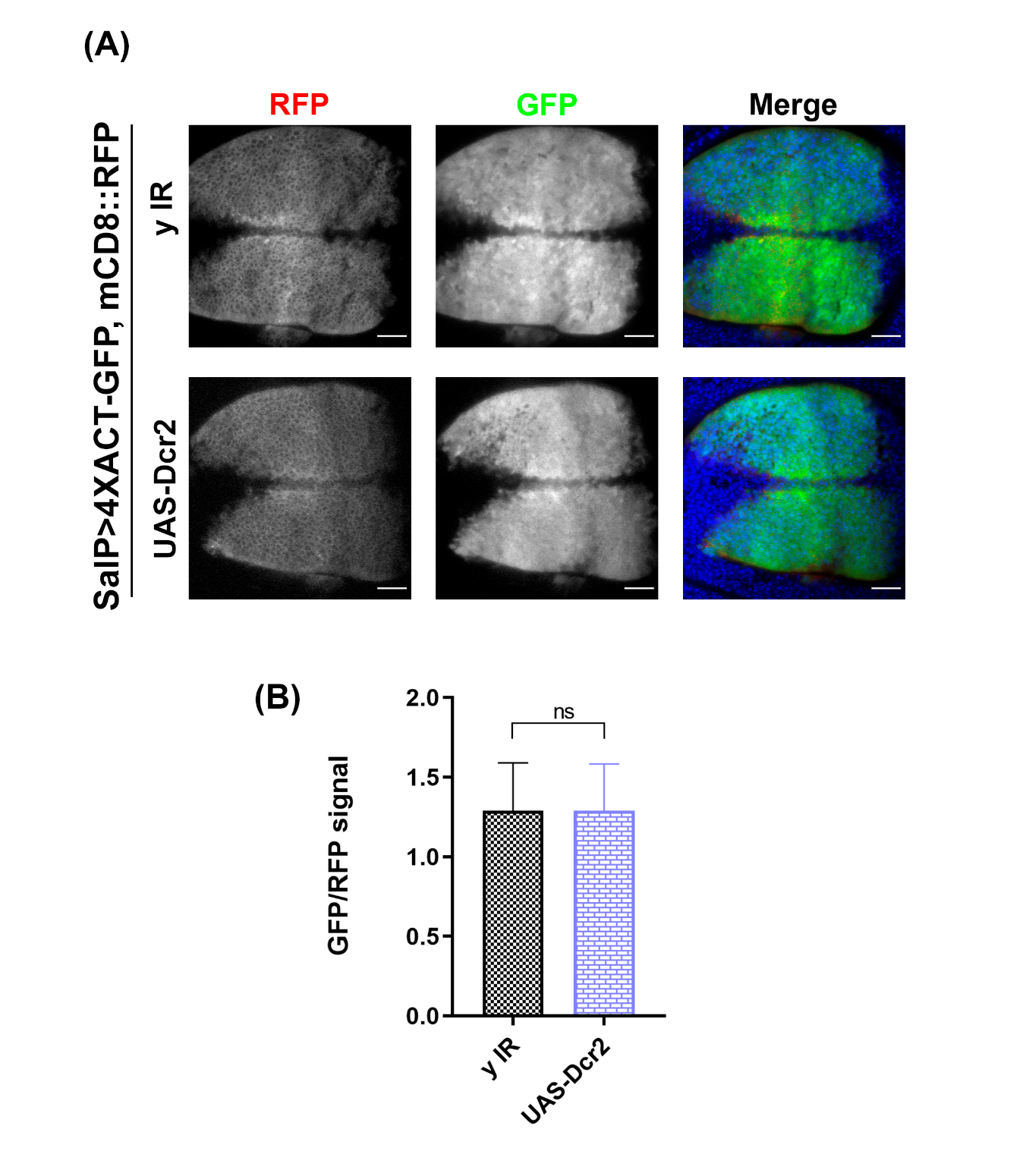


Figure S2: (A) 4XACT-GFP reporter overexpression in third instar larvae wing discs in control (RNAi against yellow) and Dcr2 overexpression. (B) Unpaired t-test shows no statistically significant difference between both conditions, p>0,05, n= 21 and 18 discs for each condition, from three independent crosses. Scale bar = 20µm.


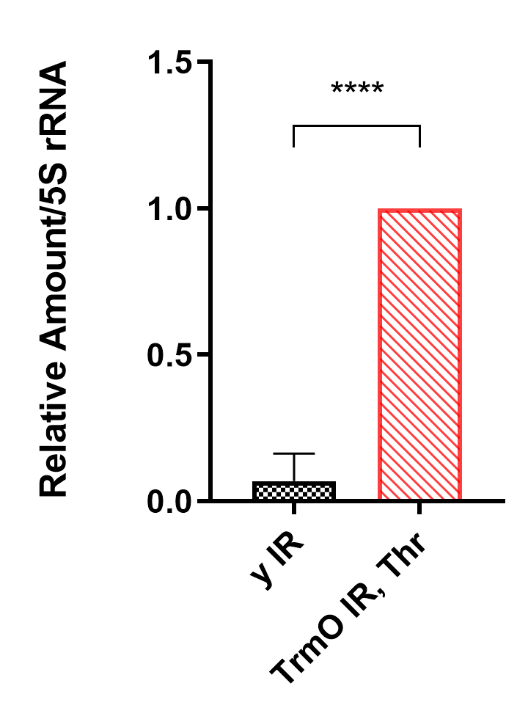


Figure S3: Overexpression of tRNA-Thr-AGT in third instar larvae. Unpaired t-test of FL-PCR targeting tRNA-Thr-AGT under overexpression conditions using our U6B construct, p<0.05, data from three different independent experiments. 5S rRNA qRT-PCR was used as a normalizer.


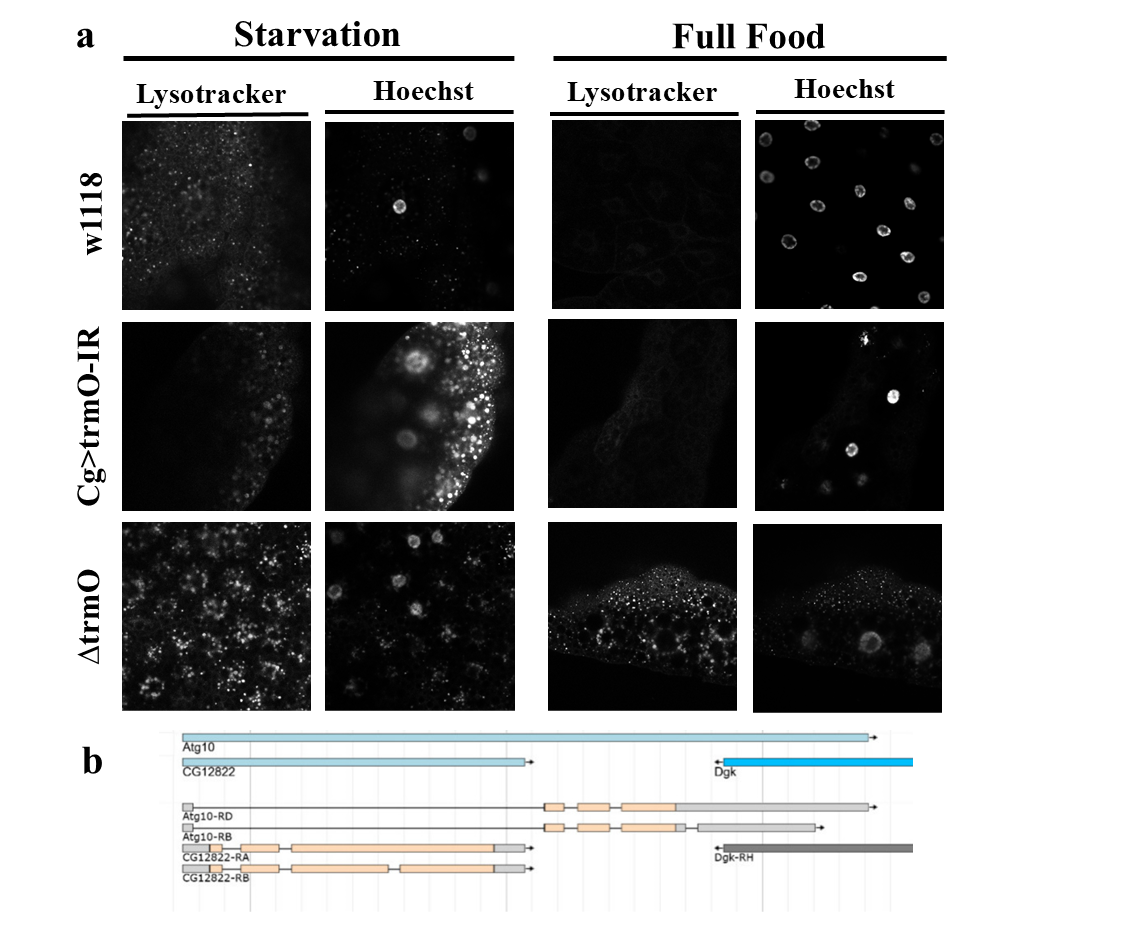


Figure S4: (a) Lysotracker and Hoescht staining in third instar larvae fat body under normal or starvation medium, see materials and methods. Results suggest that dTrmO knockdown does not impair autophagy. (b) Genomic position of dTrmO (CG12822). Source: Flybase.


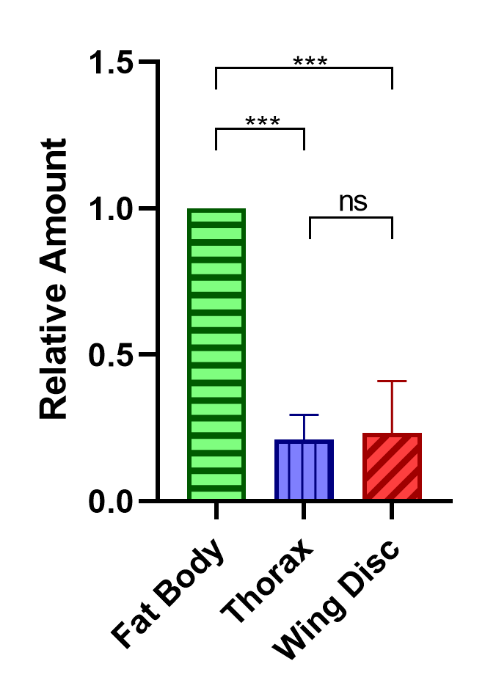


Figure S5: Fold change expression of mature tRNA^Thr^_AGT_ in the larval fat body, thorax, and imaginal wing discs normalized by 5S rRNA levels shows a significant difference between the fat body and thorax, and fat body and wing disc tRNA^Thr^_AGT_ expression. Each bar represents data from three independent experiments.


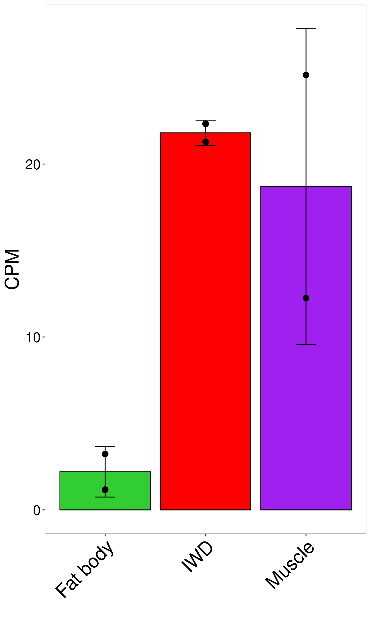


Figure S6: Expression levels of dTrmO in tissue-specific transcriptomes were calculated using Counts Per Million (CPM) across individual replicates. Bar plots represent the mean values along with standard deviations. dTrmO expression levels were quantified from the same transcriptome described in Brown et al., 2014, using the Counts Per Million (CPM) method. For each replicate, CPM was calculated as (gene read count / total reads) × 1,000,000. Mean expression levels and standard deviations were computed for each tissue.


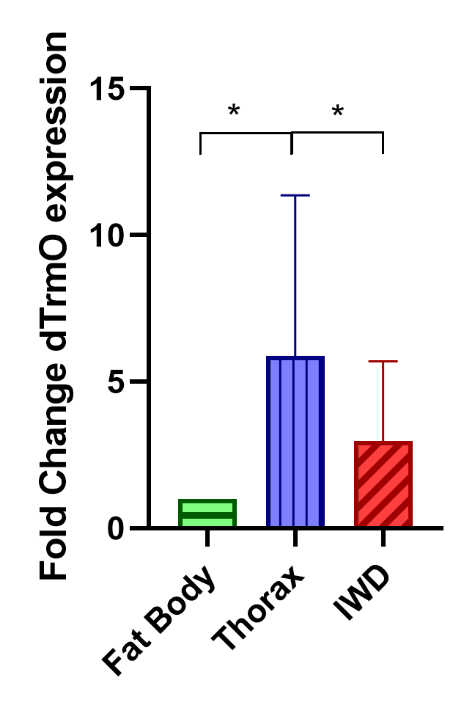


Figure S7: Tissue specific qRT-PCR experiments of dTrmO expression. Two sample comparison t-test shows differences between fat body and thorax, as well as thorax and wing discs, p<0.05.


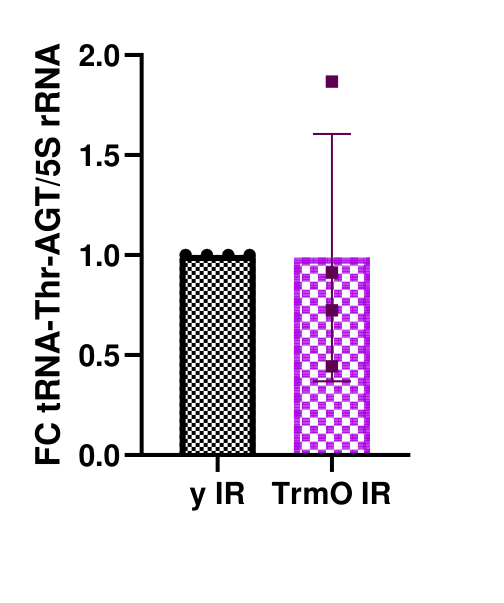


Figure S8: Relative levels of tRNA-Thr-AGT in control and dTrmO knockdown third instar larvae. T-test p < 0.05, samples come from four independent biological replicates.


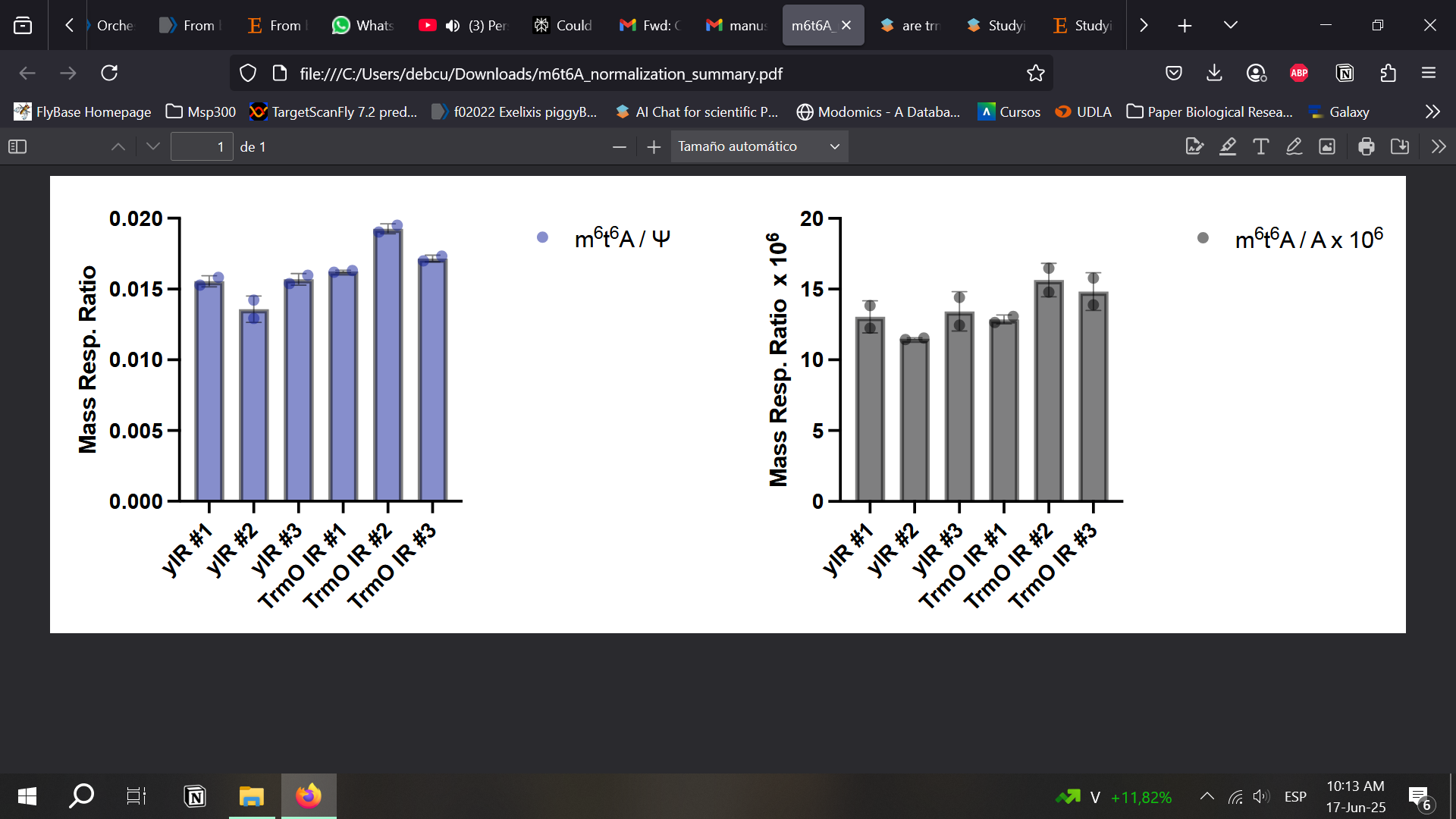


Figure S9: Total RNA nucleoside analysis of RNAi-treated whole Drosophila. Mass response ratio between m6t6A (427.1 -> 295.2) and  (245.1 -> 209.1) was displayed in bar graph with standard deviation from two technical replicates.


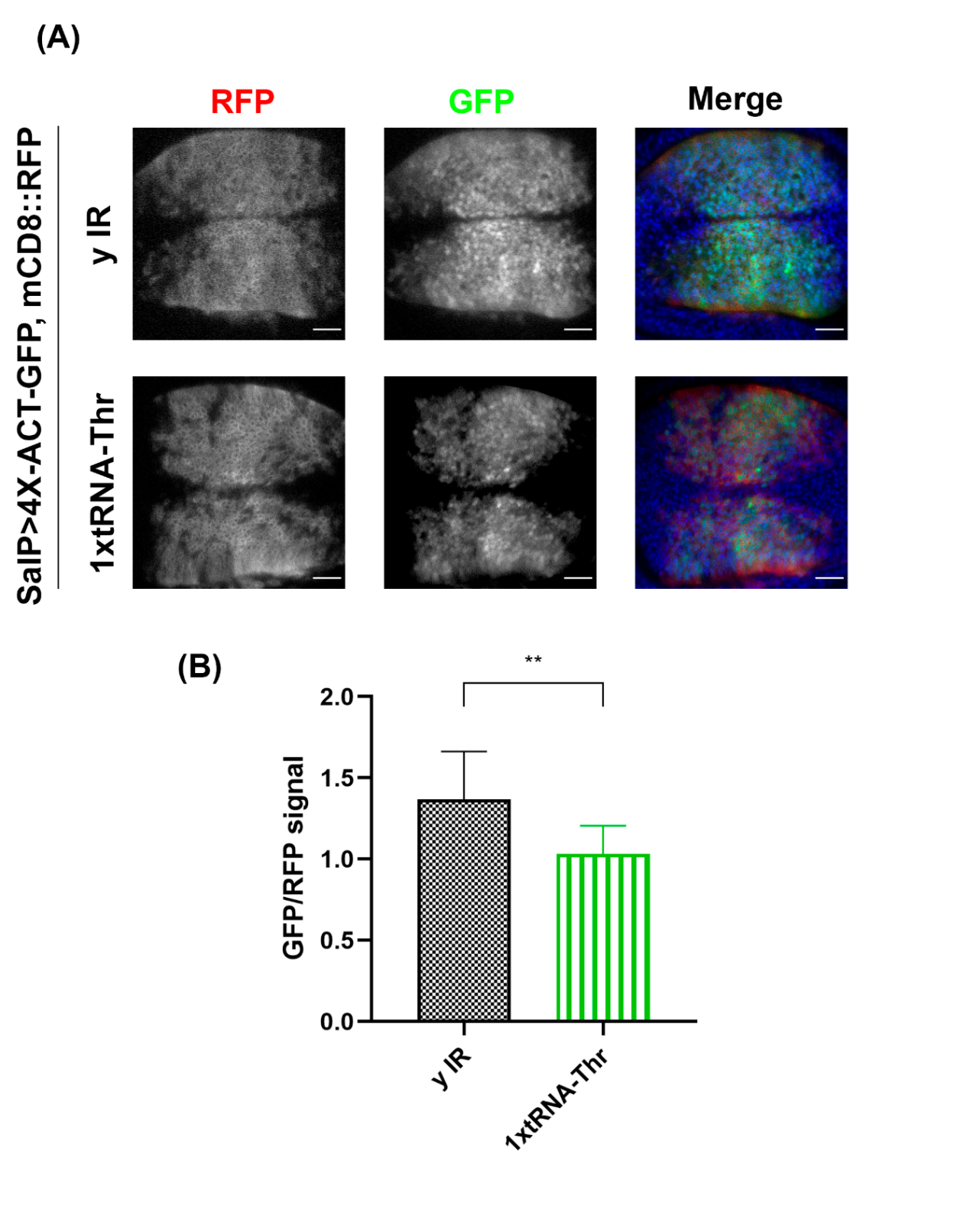


Figure S10: (A) 4X-ACT-GFP reporter overexpression in third instar larvae wing discs in control (y IR) and tRNA^Thr^_AGT_ overexpression. (B) Unpaired t-test of GFP/RFP signal shows a significant difference between controls and tRNA overexpression in wing discs (p<0,05). n = 12 discs for each condition from two independent crosses. Scale bar = 20µm.


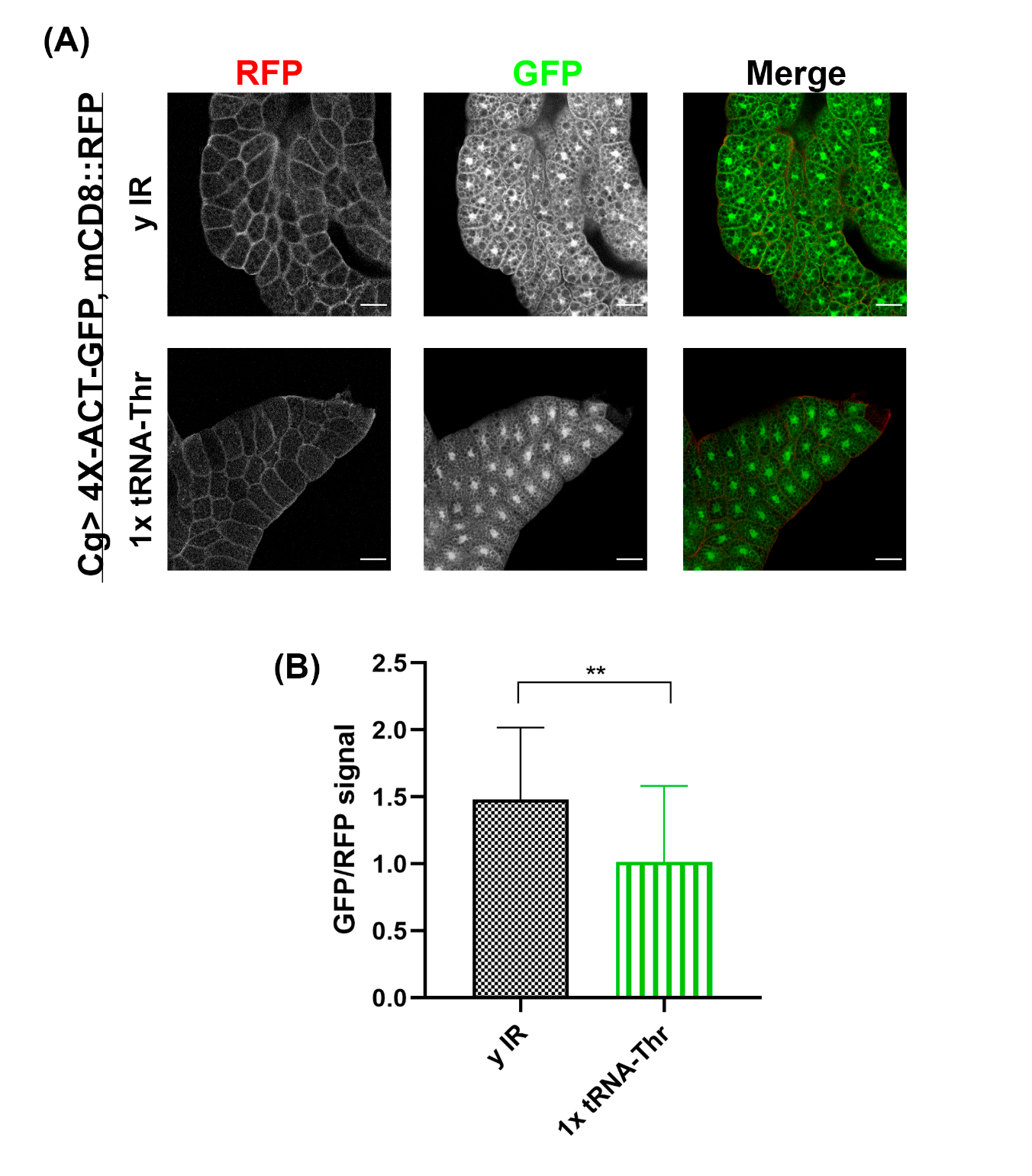


Figure S11: Translation of 4X-ACT-GFP reporter in larval fat body under overexpression of tRNA^Thr^_AGT_. (A) Fat body mounts for each condition. (B) Unpaired t-test shows a slight decrease in GFP/RFP signal under tRNA^Thr^_AGT_ overexpression. n= 36 and 29 ROI from different images of fat body mounts, from three independent crosses. Scale bar = 50µm.
